# The Value of Wise Sanitary Measures

**Published:** 1897-01

**Authors:** 


### THE VALUE OF WISE SANITARY MEASURES.

UNDER " Control Work " there will be noted some figures relative to the work of exterminating tuberculous animals in the Keystone State. This data is significant in several directions: first, it comes from a desire of our people to be rid of this great source of loss in their dairies—a determination to sell only the products of healthy herds. It gives a fair idea of the extent of the disease in suspected centres where most of these examinations have been conducted. It relieves a goodly number of herds from the danger of transmission by diseased animals, and in every instance better sanitary measures have been adopted for preservation from a fresh introduction of the disease and the spread of this as well as other infectious and contagious diseases. It has been accomplished at a relatively small cost to our people, and it has lifted a double burden from those engaged in the live-stock industry. It has strengthened the market for their products in many towns and districts of our State and given to the public increased confidence in our State officials and legislators who have provided these means for the better protection of the people within our borders. The whole tendency of the work has been educational, protective, and helpful to the people of our State without placing a burden upon any one class.

